# Predictors for Target Vessel Failure after Recanalization of Chronic Total Occlusions in Patients Undergoing Surveillance Coronary Angiography

**DOI:** 10.3390/jcm9010178

**Published:** 2020-01-09

**Authors:** Martin Geyer, Johannes Wild, Marc Hirschmann, Zisis Dimitriadis, Thomas Münzel, Tommaso Gori, Philip Wenzel

**Affiliations:** 1 Center for Cardiology, Cardiology I, University Medical Center Mainz of the Johannes Gutenberg-University Mainz, Langenbeckstr. 1, 55131 Mainz, Germany; Johannes.wild@unimedizin-mainz.de (J.W.); marc.hirschmann@icloud.com (M.H.); zisis.dimitriadis@unimedizin-mainz.de (Z.D.); tmuenzel@uni-mainz.de (T.M.); tommaso.gori@unimedizin-mainz.de (T.G.); 2 Center for Thrombosis and Hemostasis, University Medical Center Mainz of the Johannes Gutenberg-University Mainz, Langenbeckstr 1, 55131 Mainz, Germany; 3 German Center for Cardiovascular Research (DZHK), Partner Site Rhine Main, Langenbeckstr. 1, 55131 Mainz, Germany

**Keywords:** chronic total occlusion, target vessel failure, re-occlusion, surveillance coronary angiography

## Abstract

(1) Background: Knowledge about predictors for the long-time patency of recanalized chronic total coronary occlusions (CTOs) is limited. Evidence from invasive follow-up in the absence of acute coronary syndrome (routine surveillance coronary angiography) is scarce. (2) Methods: In a monocentric-retrospective analysis, we obtained baseline as well as periprocedural data of patients undergoing routine invasive follow-up. We defined target vessel failure (TVF) as a combined primary endpoint, consisting of re-occlusion, restenosis, and target vessel revascularization (TVR). (3) Results: We included 93 consecutive patients (15.1% female) from October 2013 to May 2018. After a follow-up period of 206 ± 129 days (median 185 (IQR 127–237)), re-occlusion had occurred in 7.5%, restenosis in 11.8%, and TVR in 5.4%; the cumulative incidence of TVF was 15.1%. Reduced TIMI-flow immediately after recanalization (OR for TVR: 11.0 (95% CI: 2.7–45.5), *p* = 0.001) as well as female gender (OR for TVR: 11.0 (95% CI: 2.1–58.5), *p* = 0.005) were found to be predictive for pathological angiographic findings at follow-up. Furthermore, higher blood values of high-sensitive troponin after successful revascularization were associated with all endpoints. Interestingly, neither the J-CTO score nor the presence of symptoms at the follow-up visit could be correlated to adverse angiographic results. (4) Conclusions: In this medium-sized cohort of patients with surveillance coronary angiography, we were able to identify reduced TIMI flow and female gender as the strongest predictors for future TVF.

## 1. Introduction

Coronary chronic total occlusion (CTO) is defined as either absent or minimal antegrade coronary blood flow diagnosed by coronary angiography that had existed for >12 weeks [1]. According to registry data, this distinct subtype of coronary artery disease has a prevalence of up to 20% of all invasive coronary diagnostics [2]. Nevertheless, expert opinion on the optimal treatment strategy (conservative, interventional, or surgical) is still controversial. According to contemporary data on clinical practice, only about one third of all patients with a CTO are treated by revascularization (percutaneous coronary intervention (PCI) or coronary artery bypass grafting surgery (CABG)) [2]. In contrast to non-CTO PCIs with a procedural success rate of 98%, interventional CTO procedures are more complex and have a significantly lower periprocedural revascularization rate of 60% to 70% in non-specialized centers [2,3], which can exceed 90% in highly specialized units [4,5]. As a tool to assess and grade lesion difficulty as well as predicting successful guidewire crossing within 30 min in interventional recanalization, the J-CTO (Multicenter CTO Registry in Japan) score was developed and validated. The presence of five specific lesion characteristics in CTO vessels that are known to hamper revascularization success (blunt stump, occlusion length > 20 mm, calcification, vessel bending > 45 degrees, and previously failed PCI) are assigned to one point each and summarized [6]. Vessel revascularization in CTO lesions has been associated with clinical improvement of angina and a prognostic benefit regarding a lower rate of subsequent myocardial infarction and longer survival in clinical registries [7,8,9,10]. However, evidence on long-term angiographic results as well as potential predictors for vessel patency and re-occlusion post CTO-PCI is scarce. Results from other registries imply that a higher pre-interventional J-CTO score—beyond acute success—might have an impact on an increased probability for future adverse events [11,12]. Thus, the objectives of this study were (i) to investigate the incidence of long-term target vessel failure (re-occlusion, restenosis, and target vessel revascularization) as assessed by invasive follow-up in an all-comer retrospective monocentric analysis, and (ii) to identify potential predictors of future target vessel failure after successful CTO recanalization, including the J-CTO score.

## 2. Methods

Data of all patients consecutively treated with successful PCI for CTO-lesions in our center between October 2013 to September 2017 that had an elective control coronary angiography until May 2018 were included in this retrospective analysis. Surveillance angiography after a follow-up period of 3 to 12 months was routinely recommended after successful recanalization of a CTO vessel in accordance with the guidelines for high-risk lesions [13]. Patients primarily undergoing urgent invasive control for acute coronary syndrome at a follow-up instead of the elective control coronary angiography were excluded from the analysis. From October 2013 to September 2017, recanalization of CTO lesions by PCI was successfully performed in 201 cases in our center. For this retrospective analysis, data of 100 patients of this cohort with an invasive follow-up in our center were available (49.8%). Seven patients were excluded because of either an extremely long latency from the index procedure to follow-up (*n* = 3) and/or because of acute coronary syndrome as an indication for repeated invasive coronary angiography (*n* = 5) in order to prevent potential bias by findings not solely grounded on previously recommended routine control. All subjects were adult individuals (≥18 years) with pre-existing fluoroscopic evidence of a chronically occluded coronary vessel. The choice of the interventional approach (antegrade vs. retrograde recanalization, radial or femoral access) and material for intervention at the index visit (e.g., guiding catheters, guidewires, PCI balloons, and stents) was subject to the discretion of the operator.

Patients’ characteristics, clinical features (angina pectoris: Defined as chest discomfort as classified by Canadian Cardiovascular Society (CCS) class >2; symptoms: Defined as the presence of angina pectoris CCS class >2 and/or dyspnea NYHA (New York Heart Association) class >1; echocardiographic baseline parameters; proof of vitality of the region of the CTO), comorbidities, cardiovascular risk factors, as well as features of the PCI procedure (e.g., treated vessel, dose of contrast dye and radiation, fluoroscopy and procedural duration, J-CTO score and its subfactors (lesion entry, length, bending, and previously failed PCI attempt) of the lesion, number and length of used stent material), periprocedural levels of biomarkers (e.g., high-sensitive troponin I, creatinine, C-reactive protein), and clinical and angiographic findings at the invasive follow-up visit were gathered and analyzed. For a detailed protocol to quantify the J-CTO score, see [6]. Adipositas was defined as BMI (Body mass index) ≥ 30 kg/m^2^, according to the WHO definition. Renal impairment was defined as a glomerular filtration rate < 60 mL/min*1.73m^2^. The grade of a potential restenosis at the follow-up coronary angiography was retrospectively reassessed and the diameter loss in comparison to the reference vessel diameter was quantified in a semi-automatic manner by the Quantitative Coronary Analysis (QCA) tool (Philips Healthcare, Andover, MA, USA) for this study. Additionally, TIMI flow—as defined by the Thrombolysis in Myocardial Infarction Trial, quantified in Grades 0–3—was recorded semi-quantitatively for the time points directly after the index procedure and at the follow-up visit.

The primary endpoints of the retrospective analysis of our patient cohort study were defined as follows:

(1) Re-occlusion: Defined as TIMI flow grade 0, as assessed by fluoroscopy of the treated vessel at the timepoint of surveillance coronary angiography.
(2) Restenosis: Defined as the recurrence of lumen loss >50% in the CTO vessel as quantified retrospectively by QCA (including re-occlusion).
(3) Target vessel failure (TVF): Defined as a combined endpoint by the presence of re-occlusion, restenosis, or target vessel revascularization (defined as a necessity for a repeated PCI within the former CTO vessel).

We compared baseline parameters and values of clinical, fluoroscopic, and laboratory findings during index hospitalization (CTO PCI procedure) and at the timepoint of invasive follow-up and assigned patients to groups dependent on the presence of each singular endpoint as well as the combined endpoint at the time of follow-up surveillance coronary angiography. Continuous variables are presented as a mean ± standard deviation or as a median and interquartile range and categorial variables are expressed as percentages. Continuous variables found not to follow a normal distribution when tested with the modified Kolmogorov–Smirnov test (Lilliefors test) and Shapiro–Wilk-test were compared using the Wilcoxon matched-pairs signed rank test or the Wilcoxon–Mann–Whitney test for comparison between each two groups. Normally distributed continuous variables were compared using the Students’ *t*-test and categorical variables with Fisher’s exact or Chi^2^ test, as appropriate.

Logistic regression analyses were performed in order to identify potential predictors for the occurrence of endpoints. Odds ratios (ORs) are given with the corresponding 95% confidence intervals (CIs). Logistic regressions were calculated by a univariate and a multivariate model, which was adjusted for age, diabetes, hyperlipidemia, smoking, hypertension, and a positive family history of cardiovascular disease. Receiver operating characteristics (ROCs) curves were calculated for the sensitivity and specificity of the J-CTO score to predict each individual endpoint, and the areas under the curve (AUC) are presented with the corresponding 95% CI. *p* values < 0.05 (two-sided) were considered to be statistically significant. Statistical analysis was conducted using SPSS software version 24 (SPSS Inc., Chicago, IL, USA).

Since the study involved only an anonymized, retrospective analysis of diagnostic standard data, ethics approval was not required according to German law.

## 3. Results

At the time of the index procedure, the 93 patients included in our analysis had a mean age of 65.6 ± 11.0 years old, 15.1% of them were female, and they had been symptomatic (angina or dyspnea) before intervention in 81.7% of the cases. The predominant target vessel for CTO intervention was the right coronary artery in 54.8% of the cases and the mean J-CTO score was 1.49 ± 1.09. Most predominant cardiovascular risk factors comprised arterial hypertension (79.6%), smoking (57.0%), and hyperlipidemia (59.1%) as well as a history of previous PCI (74.2%). A detailed overview of the baseline characteristics of all included subjects is displayed in Table 1. A mean of 2.2 ± 1.1 stents were implanted over an average lesion length of 56.6 ± 30.5 mm. One patient (1.1%) received treatment with a drug-eluting balloon alone without additional stent implantation; in all other patients, second-generation drug-eluting stents or scaffolds (in 76 cases (82.8%), everolimus-eluting stents (EESs); in 4 cases (4.3%), biolimus eluting stents; in 9 cases (9.7%), everolimus-eluting bioresorbable vascular scaffolds (BVSs), and in 3 cases (3.2%), a combination of EESs and BVSs) were used to treat the lesion. No patient was treated by POBA (plain old balloon angioplasty). Three patients (3.2%) encountered periinterventional acute renal failure, and one patient (1.1%) had relevant bleeding at the site of vascular access; in all other patients, no relevant major adverse events during the index visit were recorded. In total, 95.7% (*n* = 89) of the recanalizations were performed via the antegrade approach and primary vascular access was via the radial artery in 62.4% of cases (*n* = 58). In accordance with the guidelines [8], 89.2% (83 patients) were treated with dual anti-platelet therapy alone, and 10 cases (10.8%) with a combination of antiplatelet therapy and an oral anticoagulant. The time elapsed from the index procedure to invasive follow-up was, on average, 206 ± 129 days (median 185 (IQR 127–237 days)).

We compared patients’ clinical and periinterventional characteristics, including gender, coronary vascular risk factors, renal impairment, left ventricular ejection fraction, duration and cumulative fluoroscopy dose, stent length and number, periinterventional biomarkers, and symptoms, at baseline and follow-up for each individual endpoint, and the cumulative endpoint. The incidence of re-occlusion was low (7.5%, *n* = 7) and re-stenosis of the former CTO lesion (including re-occlusion) was observed in 11.8% (*n* = 11). In five patients (5.4%), TVR was performed (two patients with treatment within the former CTO lesion, in three patients with de novo stenosis adjacent to the former CTO lesion). Thus, the incidence of the combined endpoint TVF was 15.1% (*n* = 14). Detailed results are presented in Table 2 (for enhanced results, see Supplementary Materials Table S1).

When comparing baseline characteristics as well as periprocedural factors of the index procedure of patients encountering endpoints to those without adverse outcomes at the time of follow-up, we identified several parameters with statistically significant differences between the patient groups. Patients with reduced TIMI flow of the target vessel directly at the end of the index procedure were statistically significantly overrepresented in the groups encountering each of the endpoints. We observed a significantly greater incidence of re-occlusion (100% vs. 8.1%, *p* < 0.001), restenosis (90.9% vs. 4.9%, *p* < 0.001), and the combined endpoint (71.4% vs. 5.1%, *p* < 0.001). Furthermore, the patients reaching the endpoints had higher periprocedural levels of high-sensitive troponin I (1126.3 ± 1560.6 vs. 412.0 ± 1391.4, *p* = 0.006 for re-occlusion, 771.4 ± 1255.7 vs. 420.7 ± 1425.5, *p* = 0.013 for restenosis, and 662.4 ± 1124.7 vs. 425.5 ± 1451.3, *p* = 0.044 for TVF), and the cumulative length of implanted stents was significantly shorter (36.3 ± 41.1 vs. 58.3 ± 29.2, *p* = 0.044 for re-occlusion, 38.2 ± 36.2 vs. 59.1 ± 29.0 mm, *p* = 0.020 for restenosis, 43.0 ± 35.9 vs. 59.0 ± 29.0 mm, *p* = 0.042 for TVF). Other factors with statistically significant differences between the groups encountering endpoints were a lower number of implanted stents for restenosis, as well as a longer cumulative duration of the CTO index procedure both for re-occlusion and restenosis. Patients with female gender were significantly overrepresented in the target vessel failure group at follow-up (35.7% vs. 11.3%, *p* = 0.034)—a similar trend could also be observed for restenosis and re-occlusion, although this did not reach statistical significance (for details, see Table 2).

We performed logistic regression analyses to assess the odds ratios of independent predictors for each individual endpoint, including TVF, and adjusted those further for general cardiovascular risk factors (age, diabetes, hyperlipidemia, smoking, hypertension, and family history of cardiovascular disease) in a multivariate model. The results are presented in Table 3 (for further detailed calculations, see online Supplementary Materials Table S1). Individual factors as potential predictors for TVF comprised—as expected—reduced TIMI flow at the end of the index procedure all endpoints for re-occlusion (OR: 20.36 (95% CI: 3.21–129.00), *p* = 0.001), restenosis (OR: 21.29 (95% CI: 4.28–105.97), *p* < 0.01), and the combined endpoint/TVF (OR: 11.00 (95% CI: 2.66–45.45), *p* = 0.001). Female gender proved to be a predictor for the occurrence of restenosis (OR: 8.88 (95% CI: 1.58–49.89), *p* = 0.013) as well as target vessel failure (OR: 11.03 (95% CI: 2.08–58.47), *p* = 0.005). Of note, a lower BMI was assessed to be predictive regarding the endpoints of restenosis (OR: 0.73 (95% CI: 0.55–0.98), *p* = 0.037) and TVF (OR: 0.80 (95% CI: 0.65–0.99), *p* = 0.037).

**Figure 1:**
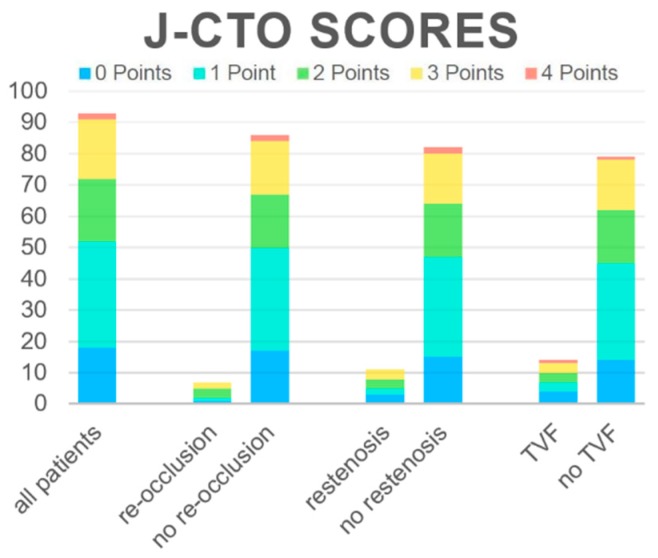
Distribution of the J-CTO score in all patients in the groups reaching endpoints. J-CTO scores were calculated for the whole study group and stratified for all single endpoints and the combined endpoint. In our study, the distribution of J-CTO scores did not differ significantly between groups (for details, see the text).

The J-CTO score at the index procedure as well as the presence of its singular factors could not be correlated with the later occurrence of any of the singular endpoints or TVF. Neither was there any statistically significant difference between the groups reaching the endpoints and those without adverse events, nor were any ORs statistically significant (Figure 1). In order to further determine the sensitivity and specificity to predict each individual end point by the J-CTO score, we computed ROC curves. The AUC for re-occlusion was calculated as 0.61 (95% CI 0.40–0.82), for restenosis as 0.52 (95% CI 0.32–0.71), and for TVF as 0.51 (95% CI 0.33–0.70) (see online Supplementary Materials Figure S1), further documenting that the J-CTO score could not predict later adverse outcomes in our cohort. Interestingly, the presence of typical angina pectoris and/or dyspnea at the time of follow-up did also not have any correlation with the co-incidence of re-occlusion, restenosis, or TVR (see Table 2).

## 4. Discussion

Over the last years, percutaneous recanalization procedures of CTO lesions have been introduced into daily clinical practice in most PCI centers. Successful intervention in CTO lesions has been attributed to clinical as well as prognostic benefit [7,8,9]. Yet, follow-up data, including invasive control coronary angiography, as well as evidence on potential predictors for long-term success are rare.

The key findings of this retrospective study are as follows: In a monocentric retrospective analysis with routinely recommended invasive follow-up of intermediate to difficult CTO lesions (mean J-CTO score 1.49 ± 1.09), re-occlusion rates tended to be low. Yet, the incidence of adverse findings, like restenosis, target lesion revascularization, and the combined endpoint target vessel failure, was moderate but still relevant. Of all clinical parameters entered in the analysis, reduced TIMI flow of the target vessel at the end of the index procedure was the strongest predictor of the endpoints at the follow-up visit. Furthermore, patients with higher periinterventional levels of high-sensitive troponin I as well as a shorter cumulative length of implanted stents were overrepresented in the groups with a later occurrence of adverse events at the timepoint of surveillance coronary angiography. Female patients were at a higher risk for TVF. Interestingly, the pre-procedural J-CTO score was not predictive of the occurrence of later restenosis, re-occlusion, or TLV in our cohort.

Other retrospective analyses have aimed to identify potential predictors for later cardiac adverse events in cohorts of patients that underwent PCI for CTO lesions. In a retrospective analysis of 249 patients with a mean (non-invasive) follow-up of 19.8 ± 13.1 months, a higher J-CTO score was found to be associated with a higher rate of major adverse cardiovascular events (MACEs) [11]. Although the baseline characteristics in this cohort were mainly comparable (age 63 ± 11 years vs. 65.6 ± 11.0 years in our study, 70.3% vs. 84.9% male, right coronary artery as the target vessel in 49.4% vs. 54.8%, J-CTO score 1.8 ± 1.0 vs. 1.49 ± 1.09), the study design was distinctly different, which might account for the controversial findings. The follow-up was also survey based without surveillance coronary angiography and the endpoints were also determined differently by MACEs (cardiovascular or unknown cause of death, myocardial infarction, TVR by PCI or CABG). In another large European multi-center retrospective analysis of a total of 1395 patients with a mean follow-up of 23 months, female sex, high J-CTO score ≥ 3, and prior PCI as well as reduced left ventricular function were found to be correlated with a higher incidence of MACEs [12].

In our analysis, we identified female gender as a risk factor for TVF. Although some registries generated evidence that women derive the same benefit from CTO-PCI as men in regard to clinical benefit [14], female gender was found to be a predictor of PCI-related complications as well as MACEs in other retrospective studies too [12,15,16]. The reason for this observation remains unclear but may include differences in the hormonal status between men and women. Yet, this finding might strengthen the recommendation on optimal patient pre-selection. This should comprise of routine use of non-invasive testing for myocardial ischemia prior to recanalization attempts, especially in female patients who appear to be at elevated risk for future TVF.

Only a very few studies have assessed the mechanisms and predictors of target vessel failure in CTO patients. In a prospective multicenter noninferiority trial comparing the use of a sirolimus-Eluting stent (SES) to an Everolimus-eluting stent (EES) on 330 patients with total coronary occlusions, the incidence of re-occlusion (2.2% in the SES vs. 1.4% in the EES group) and re-stenosis (8.0% vs. 2.1%) was distinctly lower than in our study group [17]. The follow-up rate was high, with 85% in comparison to nearly 50% in our study. Yet, a less strict definition of total coronary occlusion (estimated duration of occlusion ≥ 4 weeks) was utilized for this trial, which might partially account for the different findings. In a monocentric retrospective Korean registry on 235 patients with PCI for CTO with an invasive follow-up rate of 61.3% after 6 months, a longer occlusion length was found to be predictive for a higher incidence of TVR [16].

In our PCI center from which we recruited the study population, surveillance invasive follow-up was routinely recommended but only opted for in nearly 50% of the individuals. According to European Guidelines [13], follow-up coronary angiography might be routinely performed in high-risk coronary setups. The strategy of routine invasive follow-up is discussed controversially because of limited evidence and—in contrast to the situation in Europe—American guidelines abstain from a recommendation [18]. One prospective randomized multicenter study in Japan ((Randomized Evaluation of Routine Follow-up Coronary Angiography after Percutaneous Coronary Intervention Trial) ReACT Trial) on 700 patients could not find evidence of a clinical benefit for a general angiographic follow-up at least in a normal risk patient cohort [19]. Of course, it remains controversial whether CTO-PCIs resemble a high-risk PCI collective (not further explained in the European guidelines) and, furthermore, an impact on further clinical benefit by this strategy of an early invasive follow-up and treatment of probably non-symptomatic re-stenosis remains hypothetic up to now. Nevertheless, our study provides evidence that surveillance coronary angiography might be justified after recanalization of CTO lesions, especially in the presence of specific factors predictive of TVF. Clinical findings, such as ongoing symptoms alone, with definite exclusion of acute coronary syndrome, might not be helpful to stratify patients at risk of potential TVF.

Some limitations of our study merit consideration: First, the design is a monocentric retrospective analysis with a mid-term follow-up. Due to the observational nature of the study, the follow-up rate was only 49.8%, which might further account for a potential selection bias, which has to be taken into account in the interpretation of our results. BVS were used for treatment in some cases, which are not available anymore. Although routine surveillance invasive follow-up was recommended in all patients, symptomatic individuals could be overrepresented at the follow-up visit, as the prevalence of angina pectoris and dyspnea at the time of follow-up was higher in comparison to other registries [12]. Indication for TVR was based on individual assessment of the interventional operator and not mandatorily grounded on further non-invasive or invasive evaluation of the stenosis (e.g., measurement of fractional flow reserve, intracoronary imaging like optical coherence tomography or intravascular ultrasound) and a potential clinical and prognostic benefit of these interventions has not been studied. Larger prospective randomized studies with defined protocol, including intracoronary imaging or flow measurements, for surveillance coronary angiography would be desirable. Furthermore, patients with female gender were relevantly underrepresented (15.1%), yet at a comparable extent to most published CTO registries [11,12,14,15,16].

## 5. Conclusions

In this retrospective monocentric cohort of patients undergoing routine follow-up coronary angiography after CTO recanalization, we found evidence that reduced TIMI flow at the end of the index procedure as well as female gender could be predictors of later angiographic adverse outcome (TVF). Furthermore, patients with a shorter cumulative length of implanted stents and higher periinterventional levels of high-sensitive troponin I were overrepresented in the group of patients encountering re-occlusion, restenosis, and TVF at the timepoint of invasive follow-up. In contrast to other registers, we could not prove any correlation between the initial J-CTO score of the treated CTO lesion and the later occurrence of any of the endpoints. Remarkably, symptoms at the time of follow-up coronary angiography could not be attributed to adverse angiographic results. Based on the still relevant rate of TVF, even in populations of intermediate lesion complexity, such as ours, routine invasive follow-up after CTO procedures appears to be justified and should rather be guided by the presence of risk predictors, and not by the occurrence of angina (with the exception of acute coronary syndrome). Thus, our present work might stress a potential beneficial value of routine surveillance coronary angiography after CTO interventions, especially for females and patients with reduced TIMI flow at the end of the index procedure.

## Figures and Tables

**Table 1:** Baseline characteristics (*n* = 93).

Parameter	*n* (%)	Mean ± SD	Median (IQR)
Age at procedure (years)		65.6 ± 11.0	66.5 (58.2/74.6)
female gender	14 (15.1%)		
Angina before intervention	53 (57.0%)		
Symptoms before intervention	76 (81.7%)		
multivessel disease	80 (86.0%)		
previous CABG	10 (10.8%)		
previous PCI	70 (74.2%)		
Diabetes	31 (33.3%)		
Smoking	53 (57.0%)		
Hyperlipidemia	55 (59.1%)		
Family history of CAD	24 (25.8%)		
arterial hypertension	74 (79.6%)		
peripheral artery disease	9 (9.7%)		
cerebral artery disease	8 (8.6%)		
renal insufficiency	6 (6.4%)		
hyperthyroidism	13 (14.0%)		
weight (KG)		90.2 ± 20.3	87.3 (78.0/100.8)
height (meters)		1.74 ± 0.10	1.76 (1.68/1.80)
Body mass index (kg/m^2^)		25.8 ± 4.8	24.9 (22.8/28.4)
Adipositas	14 (21.2%)		
mean LVEF (%)		50.5 ±9.6	55.0 (45.0/55.0)
reduced LVEF at baseline	19 (29.7%)		
proof of vitality of CTO region prior to intervention	52 (57.8%)		
CTO vessel			
LAD	19 (20.4%)		
LCX	23 (24.7%)		
RCA	51 (54.8%)		
J-CTO Score		1.49 ± 1.09	1.0 (1.0/2.0)
Components of the J-CTO Score			
Entry	27 (29.0%)		
Calcification	47 (50.5%)		
Bending > 45°	25 (26.9%)		
Lesion Length > 20 mm	29 (31.2%)		
Retry Lesion	12 (12.9%)		

Abbreviations: CABG: coronary artery bypass grafting, PCI: percutaneous coronary intervention, CTO: chronic total occlusion, LVEF: left ventricular ejection fraction. LAD: left anterior descending artery, LCX: left circumflex artery, RCA: right coronary artery. IQR: Interquartile Range, SD: standard deviation.

**Table 2:** Patients’ baseline, periprocedural, and follow-up characteristics stratified for endpoints.

	Re-Occlusion			Restenosis			TVF		
	Re-Occlusion (*n* = 7)	No Re-Occlusion (*n* = 86)	*p*-Value	Restenosis (*n* = 11)	No Restenosis (*n* = 82)	*p*-Value	TVF (*n* = 14)	No TVF (*n* = 79)	*p*-Value
**Baseline parameters**									
**female gender**	28.6	14.0	0.283	36.4	12.2	0.058	35.7	11.3	0.034
**Age at procedure**	65.1 ± 6.9	65.6 ± 11.3	0.843	65.6 ± 7.5	65.6 ± 11.5	0.988	60.6 ± 13.4	66.5 ± 10.4	0.063
**Reduced LVEF**	20.0	30.5	1.000	22.2	30.9	0.713	30.0	29.6	1.000
**LVEF baseline**	51.8 ± 6.6	50.4 ± 9.8	0.923	51.0 ± 6.4	50.5 ± 10.0	0.667	50.4 ± 6.4	50.6 ± 10.1	0.466
**Angina at baseline**	57.1	57.0	1.000	54.5	57.3	1.000	50.0	58.2	0.574
**Symptoms at baseline**	100	80.2	0.342	100.0	79.3	0.206	92.9	79.7	0.453
**Body Mass Index**	23.6 ± 2.1	25.9 ± 4.9	0.356	23.1 ± 2.7	26.1 ± 4.9	0.130	23.8 ± 4.8	26.1 ± 4.8	0.158
**J-CTO Score**	1.86 ± 1.07	1.47 ± 1.09	0.307	1.55 ± 1.21	1.49 ± 1.08	0.843	1.60 ± 1.82	1.49 ± 1.05	0.889
**J-CTO Score ≥ 3**	28.6	22.1	0.654	37.5	22.0	0.707	28.6	21.5	0.511
**Periprocedural characteristics**									
**CTO vessel**									
**- LAD**	28.6	19.8	0.332	18.2	20.7	0.969	14.3	21.5	0.811
**- LCx**	42.9	23.3		27.3	24.4		28.6	24.1	
**- RCA**	28.6	57.0		54.5	54.9		57.1	54.40	
**Reduced TIMI-flow post intervention**	100.0	8.1	<0.001	90.9	4.9	<0.001	71.4	5.1	<0.001
**Stent length (mm)**	36.3 ± 41.1	58.3 ± 29.2	0.044	38.2 ± 36.2	59.1 ± 29.0	0.020	43.0 ± 35.9	59.0 ± 29.0	0.042
**Stent number**	1.6 ± 1.6	2.2 ± 1.0	0.065	1.6 ± 1.4	2.2 ± 1.0	0.040	1.9 ± 1.3	2.2 ± 1.0	0.183
Fluoroscopy dose (cgy*dm)	8062 ± 4148	7363 ± 6308	0.351	7547 ± 4697	7398 ± 6352	0.677	7134 ± 4324	7465 ± 6449	0.830
**Fluoroscopy time (min)**	29.6 ± 18.0	26.0 ± 15.9	0.570	30.8 ± 18.8	25.7 ± 15.6	0.388	30.2 ± 18.0	25.6 ± 15.6	0.347
**Duration (total) (min)**	165.1 ± 26.8	123.8 ± 44.8	0.006	154.6 ± 44.0	123.2 ± 44.0	0.013	144.4 ± 45.7	123.8 ± 44.4	0.056
**Contrast volume (mL)**	277.4 ± 159.4	240.7 ± 103.1	0.662	279.2 ± 141.2	238.8 ± 102.4	0.372	263.8 ± 132.2	239.9 ± 103.2	0.576
**Periinterventional CK (u/L)**	178.3 ± 141.2	116.0 ± 94.9	0.276	132.7 ± 120.8	118.8 ± 96.6	0.929	123.5 ± 108.9	119.9 ± 98.9	0.802
**High-sensitive Troponin I periinterventional (pg/mL)**	1126.3 ± 1560.6	412.0 ± 1391.4	0.013	771.4 ± 1255.7	420.7 ± 1425.5	0.013	662.4 ± 1124.7	425.5 ± 1451.3	0.044
**Creatinine periinterventional (mg/dL)**	0.93 ± 0.11	1.16 ± 0.97	0.412	0.96 ± 0.14	1.17 ± 0.99	0.636	0.96 ± 0.13	1.18 ± 1.01	0.580
**CrP periinterventional (mg/L)**	37.0 ± 59.5	7.77 ± 15.7	0.238	23.6 ± 47.7	8.1 ± 16.4	0.712	21.8 ± 45.6	8.1 ± 16.5	0.685
**Symptoms at follow-up**									
**Angina**	28.6	32.5	1.000	36.4	31.6	0.741	35.7	31.6	0.763
**Symptoms**	42.9	53.4	0.704	54.5	52.4	1.000	50.0	53.2	1.000

values presented as percentages or mean values ± SD. Abbreviations: LVEF: left ventricular ejection fraction, CTO: chronic total occlusion, LAD: left anterior descending artery, LCx: left circumflex artery, RCA: right coronary artery, CrP: c-reactive protein, CK: creatin kinase. TVF: Target Vessel Failure.

**Table 3:** Multivariate regression analysis (odds ratios) for re-occlusion, restenosis, and TVF.

	Re-Occlusion		Restenosis		TVF	
	OR (95% CI)	*p*-Value	OR (95% CI)	*p*-Value	OR (95% CI)	*p*-Value
**Baseline parameters**						
**female gender**	3.77 (0.54–26.43)	0.182	**8.88 (1.58–49.89)**	**0.013**	**11.03 (2.08–58.47)**	**0.005**
**Age at procedure**	0.99 (0.91–1.08)	0.822	1.00 (0.93–1.07)	0.995	0.95 (0.90–1.01)	0.080
**Reduced LVEF**	0.43 (0.04–5.09)	0.426	0.49 (0.08–3.10)	0.449	0.70 (0.13–3.87)	0.680
**LVEF baseline**	1,92 (0.91–1.16)	0.713	1.01 (0.92–1.10)	0.895	1.00 (0.92–1.09)	0.956
**Angina at baseline**	0.96 (0.19–4.79)	0.957	0.78 (0.20–2.97)	0.712	0.70 (0.21–2.40)	0.704
**Symptoms at baseline**	not calculable		not calculable		8.65 (0.62–121.31)	0.109
**Body Mass Index**	0.79 (0.57–1.09)	0.147	0.73 (0.55–0.98)	**0.037**	0.80 (0.65–0.99)	**0.037**
**J-CTO-Score**	1.42 (0.64–3.16)	0.394	1.03 (0.54–1.95)	0.929	1.11 (0.62–1.98)	0.728
**J-CTO Score ≥ 3**	1.40 (0.21–8.99)	0.721	1.26 (0.27–5.84)	0.768	1.35 (0.33–5.45)	0.676
**Periprocedural characteristics**						
**CTO vessel**	0.50 (0.18–1.38)	0.180	0.98 (0.37–2.14)	0.797	0.97 (0.43–2.19)	0.936
**-LAD**						
**-LCx**						
**-RCA**						
**Reduced TIMI-flow post intervention**	**20.36 (3.21–129.00)**	**0.001**	**21.29 (4.28–105.97)**	**<0.001**	**11.00 (2.66–45.45)**	**0.001**
**Stent length (mm)**	0.97 (0.94–1.00)	0.081	0.98 (0.95–1.00)	0.051	0.98 (0.95–1.00)	0.060
**Stent number**	0.52 (0.21–1.29)	0.156	0.58 (0.28–1.17)	0.125	0.70 (0.38–1.29)	0.255
Fluoroscopy dose (cgy*dm)	1.00 (1.00–1.00)	0.748	1.00 (1.00–1.00)	0.871	1.00 (1.00–1.00)	0.868
**Fluoroscopy time (min)**	1.02 (0.97–1.07)	0.470	1.02 (0.99–1.07)	0.233	1.03 (0.99–1.06)	0.185
**Duration (total) (min)**	**1.02 (1.00–1.04)**	**0.025**	**1.02 (1.00–1.03)**	**0.030**	1.01 (1.00–1.03)	0.056
**Contrast volume (mL)**	1.00 (1.00–1.01)	0.287	1.00 (1.00–1.01)	0.200	1.00 (1.00–1.01)	0.486
**Periinterventional CK (u/L)**	1.01 (1.00–1.01)	0.153	1.00 (0.99–1.01)	0.757	1.00 (0.99–1.01)	0.973
**High-sensitive Troponin I periinterventional (pg/mL)**	1.00 (1.00–1.00)	0.286	1.00 (1.00–1.00)	0.247	1.00 (1.00–1.00)	0.459
**Creatinine periinterventional (mg/dL)**	0.07 (0.00–8.94)	0.284	0.12 (0.00–4.57)	0.256	0.22 (0.01–5.91)	0.366
**CrP periinterventional (mg/L)**	1.03 (1.00–1.06)	0.049	1.02 (1.00–1.05)	0.067	1.02 (1.00–1.04)	0.103
**Symptoms at follow-up**						
**Angina**	0.75 (0.13–4.45)	0.750	1.24 (0.30–5.07)	0.762	0.99 (0.28–3.59)	0.992
**Symptoms**	0.69 (0.14–3.36)	0.642	1.27 (0.34–4.71)	0.723	0.92 (0.28–3.01)	0.891

Abbreviations: LVEF: left ventricular ejection fraction, CTO: chronic total occlusion, LAD: left anterior descending artery, LCx: left circumflex artery, RCA: right coronary artery, CrP: c-reactive protein, CK: creatin kinase. TVF: Target Vessel Failure.

## Supplementary Materials

The following are available online at https://www.mdpi.com/2077-0383/9/1/178/s1, Figure S1: ROC (receiver operating characteristics) curves for the J-CTO score vs. endpoints, Table S1: Detailed results for baseline, periprocedural, and follow-up data as well as uni- and multivariate regressions analysis stratified for the incidence of endpoints.
