# Supplementary material for: Predictors for Target Vessel Failure after Recanalization of Chronic Total Occlusions in Patients Undergoing Surveillance Coronary Angiography
Source: J Clin Med. 2020 Jan 9;9(1):178. doi: 10.3390/jcm9010178 (PMC7019748; doi:10.3390/jcm9010178)
Supplement: Supplementary file 1 [file jcm-09-00178-s001.pdf]

Online supplement figure 1 ROC-curves for the J-CTO Score vs. Endpoints

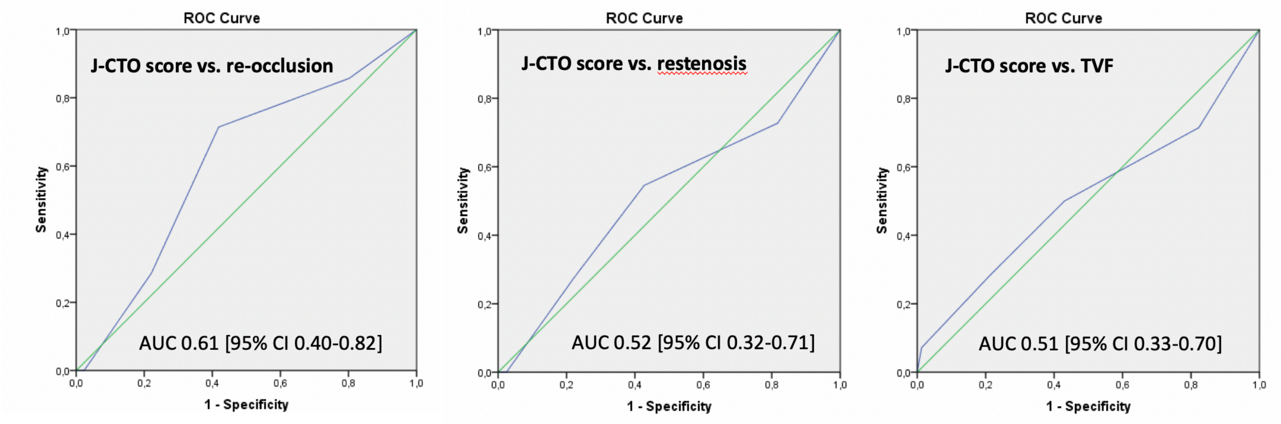

S1 Fig. 1. ROC-curves for the J-CTO Score vs. Endpoints. ROC-Curves calculated to assess sensitivity and specifity of the J-CTO score to predict the individual and the combined end points. All curves are near to the diagonal line and areas under the curve (AUC presented with 95% CI) are near 0.5, thus a relevant predictive value of the J-CTO score for adverse outcome at follow-up could not be documented in our cohort.

Online supplement Table S1: detailed results for baseline, periprocedural and follow-up data as well as uni- and multivariate regressions analysis stratified for the incidence of endpoints

S1a: re-occlusion

	Re-occlusion	no re-occlusion	p value	Univariate OR [95% CI]	P for univariate OR	Multivariate OR [95% CI]	P for multivariate OR
J-CTO Score and subfactors							
J-CTO Score	1.86±1.07	1.47±1.09	0.307	1.39 (0.69-2.80)	0.363	1.42 (0.64-3.16)	0.394
blunt stump	28.6	29.1	1.000	0.98 (0.18-5.37)	0.987	1.21 (0.20-7.15)	0.835
Calcification	71.4	48.8	0.435	2.62 (0.48-12.24)	0.265	3.00 (0.52-17.26)	0.219
Bending > 45%	42.9	25.6	0.381	2.18 (0.45-10.52)	0.331	2.04 (0.37-11.17)	0.406
Length > 20 mm	42.9	30.2	0.673	1.73 (0.36-8.29)	0.492	1.46 (0.27-8.05)	0.663
retry lesion	0.0	14.0	0.589	not calculable		not calculable	
J-CTO SUM ≥ 2	71.4	41.8	0.234	3.47 (0.64-18.91)	0.150	3.75 (0.61-22.90)	0.153
J-CTO SUM ≥ 3	28.6	22.1	0.654	1.41 (0.25-7.86)	0.695	1.40 (0.21-8.99)	0.721
Baselineparameters							
female gender	28.6	14.0	0.283	2.47 (0.43-14.19)	0.312	3.77 (0.54-26.43)	0.182
Age at procedure	65.1±6.9	65.6±11.3	0.843	1.00 (0.93-1.07)	0.891	0.99 (0.91-1.08)	0.822
Reduced LVEF	20.0	30.5	1.000	0.57 (0.06-5.46)	0.625	0.43 (0.04-5.09)	0.426
LVEF at baseline	51.8±6.6	50.4±9.8	0.923	1.02 (0.92-1.13)	0.756	1,92 (0.91-1.16)	0.713
Body mass index [kg/m²]	23.6±2.1	25.9±4.9	0.356	0.89 (0.70-1.13)	0.349	0.79 (0.57-1.09)	0.147
Adipositas	0.0	22.6	0.571	not calculable		not calculable	
Diabetes	42.9	32.6	0.682	1.55 (0.33-7.42)	0.581	1.63 (0.32-8.41)	0.560
smoking	57.1	57.0	1.000	1.01 (0.21-4.78)	0.993	0.95 (0.18-5.12)	0.948
Hyperlipidaemia	71.4	58.1	0.696				
Hypertonus	85.7	79.1	1.000	1.59 (0.18-14.05)	0.677	1.38 (0.14-13.94)	0.787
pos. familiy history	42.9	24.4	0.369				
Angina at baseline	57.1	57.0	1.000	1.01 (0.21-4.78)	0.993	0.96 (0.19-4.79)	0.957
Symptoms at baseline	100	80.2	0.342	not calculable		not calculable	
renal Insuffiency	14.3	5.8	0.383	2.70 (0.27-26.97)	0.398	2.72 (0.23-32.34)	0.427
Proof of vitality	83.3	56.0	0.395	3.94 (0.44-35.16)	0.220	3.92 (0.37-40.86)	0.254
CTO vessel	LAD 28.6, LCx 42.9, RCA 28.6	LAD 19.8, LCx 23.3, RCA 57.0	0.332	0.58 (0.23-1.45)	0.246	0.50 (0.18-1.38)	0.180
procedural characteristics							
Reduced TIMI-flow post intervention	100.0	8.1	**<0.001**	21.39 (3.51-126.74)	**0.001**	20.36 (3.21-129.00)	**0.001**
Stent length [mm]	36.3±41.1	58.3±29.2	**0.044**	0.97 (0.94-1.00)	0.082	0.97 (0.94-1.00)	0.081
Stent number	1.6±1.6	2.2±1.0	0.065	0.52 (0.22-1.24)	0.140	0.52 (0.21-1.29)	0.156
Fluoroscopy dose [cgy*dm]	8062±4148	7363±6308	0.351	1.00 (1.00-1.00)	0.772	1.00 (1.00-1.00)	0.748
Fluoroscopy time [min]	29.6±18.0	26.0±15.9	0.570	1.01 (0.97-1.06)	0.571	1.02 (0.97-1.07)	0.470

Duration (total) [min]	165.1±26.8	123.8±44.8	**0.006**	1.02 (1.00-1.04)	**0.028**	1.02 (1.00-1.04)	**0.025**
Contrast volume [ml]	277.4±159.4	240.7±103.1	0.662	1.00 (1.00-1.01)	0.387	1.00 (1.00-1.01)	0.287
Periinterventional CK [u/l]	178.3±141.2	116.0±94.9	0.276	1.00 (1.00-1.01)	0.160	1.01 (1.00-1.01)	0.153
High-sensitive Troponin I periinterventional [pg/ml]	1126.3±1560.6	412.0±1391.4	**0.013**	1.00 (1.00-1.00)	0.273	1.00 (1.00-1.00)	0.286
Creatinine periinterventional [mg/dl]	0.93±0.11	1.16±0.97	0.412	0.15(0.02-9.98)	0.372	0.07 (0.00-8.94)	0.284
CrP periinterventional [mg/l]	37.0±59.5	7.77±15.7	0.238	1.03 (1.00-1.05)	**0.038**	1.03 (1.00-1.06)	**0.049**
Follow-up visit							
Angina	28.6	32.5	1.000	0.83 (0.15-4.56)	0.830	0.75 (0.13-4.45)	0.750
Symptoms	42.9	53.4	0.704	0.65 (0.14-3.09)	0.590	0.69 (0.14-3.36)	0.642

S1b: restenosis

	restenosis	no restenosis	p value	Univariate OR [95% CI]	P for univariate OR	Multivariate OR [95% CI]	P for multivariate OR
J-CTO Score and subfactors							
J-CTO Score	1.55±1.21	1.49±1.08	0.843	1.05 (0.59-1.87)	0.868	1.03 (0.54-1.95)	0.929
blunt stump	18.2	30.5	0.500	0.51 (0.10-2.52)	0.406	0.52 (0.10-2.81)	0.449
Calcification	63.6	48.8	0.523	1.84 (0.50-6.76)	0.360	1.91 (0.48-7.64)	0.358
Bending > 45%	36.4	25.6	0.479	1.66 (0.44-6.24)	0.453	1.79 (0.42-7.61)	0.429
Length > 20 mm	36.4	30.5	0.735	1.30 (0.35-4.86)	0.693	1.14 (0.27-4.75)	0.859
retry lesion	0.0	14.6	0.348	not calculable		not calculable	
J-CTO SUM ≥ 2	54.5	42.7	0.527	1.61 (0.46-5.71)	0.460	1.64 (0.41-6.50)	0.481
J-CTO SUM ≥ 3	37.5	22.0	0.707	1.33 (0.32-5.55)	0.693	1.26 (0.27-5.84)	0.768
Baselineparameters							
female gender	36.4	12.2	0.058	4.11 (1.02-16.61)	**0.047**	8.88 (1.58-49.89)	**0.013**
Age at procedure	65.6±7.5	65.6±11.5	0.988	1.00 (0.94-1.06)	0.988	1.00 (0.93-1.07)	0.995
Reduced LVEF	22.2	30.9	0.713	0.64 (0.12-3.40)	0.599	0.49 (0.08-3.10)	0.449
LVEF at baseline	51.0±6.4	50.5±10.0	0.667	1.01 (0.93-1.09)	0.873	1.01 (0.92-1.10)	0.895
Body mass index [kg/m²]	23.1±2.7	26.1±4.9	0.129	0.86 (0.71-1.05)	0.130	0.73 (0.55-0.98)	0.037
Adipositas	0.0	23.7	0.330	not calculable		not calculable	
Diabetes	54.5	30.5	0.171	2.74 (0.76-9.81)	0.122	3.10 (0.80-12.08)	0.103
smoking	54.5	57.3	1.000	0.89 (0.25-3.17)	0.862	0.90 (0.23-3.55)	0.874
Hyperlipidaemia	54.5	59.8	0.754	0.81 (0.23-2.87)	0.742	0.69 (0.18-2.67)	0.587
Hypertonus	81.8	79.3	1.000	1.18 (0.23-5.96)	0.844	0.95 (0.16-5.55)	0.955
pos. familiy history	36.4	24.4	0.466	1.77 (0.47-6.68)	0.399	2.20 (0.53-9.09)	0.275
Angina at baseline	54.5	57.3	1.000	0.89 (0.25-3.17)	0.862	0.78 (0.20-2.97)	0.712
Symptoms at baseline	100.0	79.3	0.206	not calculable		not calculable	
renal Insuffiency	9.1	6.1	0.541	1.54 (0.16-14.55)	0.706	1.52 (0.14-16.61)	0.732
Proof of vitality	80.0	55.0	0.181	3.27 (0.65-16.39)	0.149	3.49 (0.63-19.37)	0.153
CTO vessel	LAD 18.2, LCx 27.3, RCA 54.5	LAD 20.7, LCx 24.4, RCA 54.9	0.969	1.04 (0.47-2.30)	0.931	0.98 (0.37-2.14)	0.797
procedural characteristics							
Reduced TIMI-flow post intervention	90.9	4.9	**<0.001**	18.75 (4.39-80.12)	**<0.001**	21.29 (4.28-105.97)	**<0.001**
Stent length [mm]	38.2±36.2	59.1±29.0	**0.020**	0.98 (0.95-1.00)	**0.041**	0.98 (0.95-1.00)	0.051
Stent number	1.6±1.4	2.2±1.0	**0.040**	0.55 (0.28-1.10)	0.090	0.58 (0.28-1.17)	0.125
Fluoroscopy dose [cgy*dm]	7547±4697	7398±6352	0.677	1.00 (1.00-1.00)	0.939	1.00 (1.00-1.00)	0.871
Fluoroscopy time [min]	30.8±18.8	25.7±15.6	0.388	1.02 (0.98-1.06)	0.320	1.02 (0.99-1.07)	0.233
Duration (total) [min]	154.6±44.0	123.2±44.0	**0.013**	1.01 (1.00-1.03)	**0.036**	1.02 (1.00-1.03)	**0.030**
Contrast volume [ml]	279.2±141.2	238.8±102.4	0.372	1.00 (1.00-1.01)	0.245	1.00 (1.00-1.01)	0.200

Periinterventional CK [u/l]	132.7±120.8	118.8±96.6	0.929	1.00 (1.00-1.01)	0.676	1.00 (0.99-1.01)	0.757
High-sensitive Troponin I periinterventional [pg/ml]	771.4±1255.7	420.7±1425.5	**0.013**	1.00 (1.00-1.00)	0.472	1.00 (1.00-1.00)	0.247
Creatinine periinterventional [mg/dl]	0.96±0.14	1.17±0.99	0.636	0.34 (0.02-6.87)	0.482	0.12 (0.00-4.57)	0.256
CrP periinterventional [mg/l]	23.6±47.7	8.1±16.4	0.712	1.02 (1.00-1.04)	0.111	1.02 (1.00-1.05)	0.067
Follow-up visit							
Angina	36.4	31.6	0.741	1.23 (0.33-4.61)	0.754	1.24 (0.30-5.07)	0.762
Symptoms	54.5	52.4	1.000	1.09 (0.31-3.85)	0.895	1.27 (0.34-4.71)	0.723

S1c: TVF

	TVF	No TVF	p value	Univariate OR [95% CI]	P for univariate OR	Multivariate OR [95% CI]	P for multivariate OR
J-CTO Score and subfactors							
J-CTO Score	1.60±1.82	1.49±1.05	0.889	1.08 (0.64-1.82)	0.774	1.11 (0.62-1.98)	0.728
blunt stump	21.4	30.4	0.750	0.63 (0.16-2.44)	0.499	0.64 (0.15-2.77)	0.635
Calcification	57.1	49.4	0.773	1.37 (0.43-4.31)	0.593	1.67 (0.49-5.72)	0.418
Bending > 45%	35.7	25.3	0.514	1.64 (0.49-5.47)	0.422	1.86 (0.48-7.21)	0.368
Length > 20 mm	35.7	30.4	0.757	1.27 (0.39-4.20)	0.692	1.09 (0.29-4.08)	0.904
retry lesion	7.1	13.9	0.685	0.48 (0.06-4.01)	0.494	0.40 (0.04-4.03)	0.437
J-CTO SUM ≥ 2	50.0	43.0	0.772	1.32 (0.43-4.13)	0.629	1.47 (0.40-5.37)	0.559
J-CTO SUM ≥ 3	28.6	21.5	0.511	1.46 (0.41-5.24)	0.562	1.35 (0.33-5.45)	0.676
Baselineparameters							
female gender	35.7	11.3	**0.034**	4.32 (1.18-15.78)	**0.027**	11.03 (2.08-58.47)	**0.005**
Age at procedure	60.6±13.4	66.5±10.4	0.063	0.96 (0.91-1.00)	0.071	0.95 (0.90-1.01)	0.080
Reduced LVEF	30.0	29.6	1.000	1.02 (0.23-4.44)	0.981	0.70 (0.13-3.87)	0.680
LVEF at baseline	50.4±6.4	50.6±10.1	0.466	1.00 (0.93-1.07)	0.962	1.00 (0.92-1.09)	0.956
Body mass index [kg/m²]	23.8±4.8	26.1±4.8	0.158	0.89 (0.76-1.05)	0.158	0.80 (0.65-0.99)	**0.037**
Adipositas	10.0	23.2	0.675	0.37 (0.04-3.18)	0.363	0.22 (0.02-2.17)	0.195
Diabetes	42.9	31.6	0.540	1.62 (0.51-5.17)	0.415	2.22 (0.62-7.99)	0.220
smoking	57.1	57.0	1.000	1.01 (0.32-3.18)	0.990	0.76 (0.22-2.60)	0.655
Hyperlipidaemia	50.0	60.8	0.558	0.65 (0.21-2.02)	0.453	0.74 (0.21-2.62)	0.646
Hypertonus	78.6	79.7	1.000	0.93 (0.23-3.74)	0.920	1.05 (0.24-4.68)	0.948
pos. familiy history	35.7	24.1	0.343	1.75 (0.52-5.88)	0.362	1.87 (0.49-6.49)	0.381
Angina at baseline	50.0	58.2	0.574	0.72 (0.23-2.24)	0.568	0.70 (0.21-2.40)	0.704
Symptoms at baseline	92.9	79.7	0.453	3.30 (0.40-27.14)	0.266	8.65 (0.62-121.31)	0.109
renal Insuffiency	7.1	6.3	1.000	1.14 (0.12-10.55)	0.909	1.55 (0.15-16.12)	0.713
Proof of vitality	69.2	55.8	0.545	1.78 (0.50-6.28)	0.370	2.54 (0.59-10.98)	0.212
CTO vessel	LAD 14.3, LCx 28.6, RCA 57.1	LAD 21.5, LCx 24.1, RCA 54.4	0.811	1.18 (0.56-2.47)	0.667	0.97 (0.43-2.19)	0.936
procedural characteristics							
Reduced TIMI-flow post intervention	71.4	5.1	**<0.001**	10.29 (2.79-37.88)	**<0.001**	11.00 (2.66-45.45)	**0.001**
Stent length [mm]	43.0±35.9	59.0±29.0	**0.042**	0.98 (0.96-1.00)	0.076	0.98 (0.95-1.00)	0.060
Stent number	1.9±1.3	2.2±1.0	0.183	0.72 (0.40-1.27)	0.249	0.70 (0.38-1.29)	0.255
Fluoroscopy dose [cgy*dm]	7134±4324	7465±6449	0.830	1.00 (1.00-1.00)	0.852	1.00 (1.00-1.00)	0.868
Fluoroscopy time [min]	30.2±18.0	25.6±15.6	0.347	1.02 (0.98-1.05)	0.321	1.03 (0.99-1.06)	0.185
Duration (total) [min]	144.4±45.7	123.8±44.4	0.056	1.01 (1.00-1.02)	0.120	1.01 (1.00-1.03)	0.056

Contrast volume [ml]	263.8±132.2	239.9±103.2	0.576	1.00 (1.00-1.01)	0.444	1.00 (1.00-1.01)	0.486
Periinterventional CK [u/l]	123.5±108.9	119.9±98.9	0.802	1.00 (1.00-1.01)	0.902	1.00 (0.99-1.01)	0.973
High-sensitive Troponin I periinterventional [pg/ml]	662.4±1124.7	425.5±1451.3	**0.044**	1.00 (1.00-1.00)	0.581	1.00 (1.00-1.00)	0.459
Creatinine periinterventional [mg/dl]	0.96±0.13	1.18±1.01	0.580	0.30 (0.02-4.62)	0.389	0.22 (0.01-5.91)	0.366
CrP periinterventional [mg/l]	21.8±45.6	8.1±16.5	0.685	1.02 (1.00-1.04)	0.135	1.02 (1.00-1.04)	0.103
Follow-up visit							
Angina	35.7	31.6	0.763	1.20 (0.36-3.98)	0.761	0.99 (0.28-3.59)	0.992
Symptoms	50.0	53.2	1.000	0.88 (0.28-2.75)	0.881	0.92 (0.28-3.01)	0.891
